# Patient-specific rods in adult spinal deformity: a systematic review

**DOI:** 10.1007/s43390-023-00805-8

**Published:** 2024-01-24

**Authors:** Bryce Picton, Lauren E. Stone, Jason Liang, Sean S. Solomon, Nolan J. Brown, Sophia Luzzi, Joseph A. Osorio, Martin H. Pham

**Affiliations:** 1 School of Medicine, University of California, Irvine, 101 The City Dr, Orange, CA 92868 USA; 2 Department of Neurological Surgery, University of California, San Diego, La Jolla, CA USA

**Keywords:** Patient-specific rods, Spinal deformity, Operative planning, Machine learning

## Abstract

**Purpose:**

The purpose of this review was to evaluate the effectiveness of patient-specific rods for adult spinal deformity.

**Methods:**

A systematic review of the literature was performed through an electronic search of the PubMed, Scopus, and Web of Science databases. Human studies between 2012 and 2023 were included. Sample size, sagittal vertical axis (SVA), pelvic incidence–lumbar lordosis (PI-LL), pelvic tilt (PT), operation time, blood loss, follow-up duration, and complications were recorded for each study when available.

**Results:**

Seven studies with a total of 304 adult spinal deformity patients of various etiologies were included. All studies reported SVA, and PT; two studies did not report PI-LL. Four studies reported planned radiographic outcomes. Two found a significant association between preoperative plan and postoperative outcome in all three outcomes. One found a significant association for PI-LL alone. The fourth found no significant associations. SVA improved in six of seven studies, PI-LL improved in all five, and three of seven studies found improved postoperative PT. Significance of these results varied greatly by study.

**Conclusion:**

Preliminary evidence suggests potential benefits of PSRs in achieving optimal spino-pelvic parameters in ASD surgery. Nevertheless, conclusions regarding the superiority of PSRs over traditional rods must be judiciously drawn, given the heterogeneity of patients and study methodologies, potential confounding variables, and the absence of robust randomized controlled trials. Future investigations should concentrate on enhancing preoperative planning, standardizing surgical methodologies, isolating specific patient subgroups, and head-to-head comparisons with traditional rods to fully elucidate the impact of PSRs in ASD surgery.

## Introduction

Adult spinal deformity is defined as abnormal spinal alignment from degeneration, iatrogenic developmental, or trauma in patients greater than 18 years of age [1, 2]. It is a common condition—with some studies estimating prevalence is as high as 32%—and is likely to increase as the population aged 65 and above grow [3]. The goal of ASD surgery is to reconstruct and realign the spine via a combination of osteotomies, interbody cages, instrumentation, and fusion. Traditionally, rods are contoured at the surgeon’s discretion prior to insertion, which may over or undershoot the actual necessary correction [4].

Machine learning technology created a boom in spine predictive analytics, allowing customized spinal implants to become a feasible manufacture. Beginning with pre-operative radiographs, proprietary planning software calculates specific rod parameters to meet post-operative alignment goals defined by the surgeon’s operative plan and, with new data added after each case into the machine learning model, the software is able to create more accurate plans over time [5, 6]. Specifically, this process enables the understanding of how each particular PSR geometry will influence thoracic and pelvic reciprocal changes above and below the rod post-operatively in an individual patient. These patient-specific rods (PSRs) are personalized to each patient’s optimal deformity correction.

As the prominence of patient-specific instrumentation grows, studies with appropriate follow-up are beginning to appear in the literature. However, no comprehensive systematic review of PSRs exists in the literature to date. The primary purpose of this study is to systematically review the current literature for PSRs in ASD to evaluate the capability of the technology to match planned alignment goals in adult spinal deformity surgery.

## Methods

**Fig. 1:**
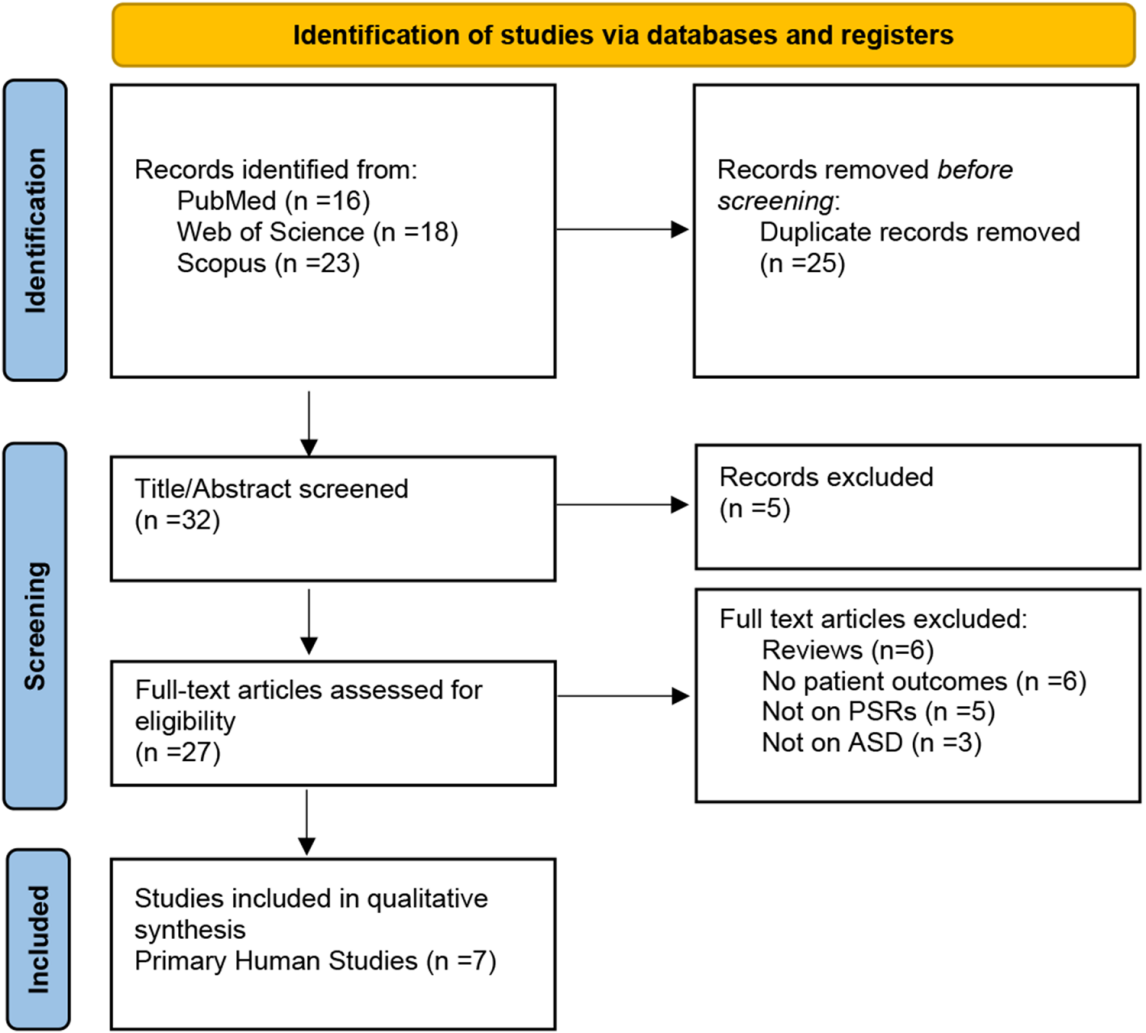
PRISMA diagram depicting the literature search process

Search queries were performed in PubMed, Web of Science, and Scopus databases using the following Boolean terms: (patient-specific rods OR patient-specific spinal rods) AND (adult spinal deformity OR ASD). The Preferred Reporting Items for Systematic Review Meta-Analyses (PRISMA) guidelines were followed. We included all primary studies published from 2012 to 2023 involving the application of PSRs for the treatment of ASD. Articles were excluded if the studies were not primary sources or patient outcomes were not reported. Information extracted from each study included author, institution and country of origin, publication year, number of subjects, and patient spinal alignment outcomes including the sagittal vertical axis (SVA), pelvic tilt (PT), and pelvic incidence–lumbar lordosis (PI-LL) difference. The literature review flow diagram is demonstrated in Fig. 1. The quality of all included studies was assessed using the Risk of Bias in Non-Randomized Studies of Interventions assessment.

## Results

### Overview of included studies

The initial search returned 57 studies. 25 duplicates were manually identified using Rayyan and excluded. The remaining 32 studies were subjected to screening by title and abstract, after which 5 studies were excluded. In the final stage of screening, 27 full-text articles were assessed for eligibility, and 20 were excluded. The majority of papers were excluded for being secondary literature (*n* = 6) and for not including quantitative outcomes (*n* = 6) (Fig. 1). Seven primary studies on the use of PSRs for the treatment of ASD included in the final review.

### Overview of results

A total of 304 patients were included in the 7 studies. Four studies were conducted in the United States between 2016 and 2020 and three in France between 2020 and 2022. All studies reported SVA, and PT; two studies did not report PI-LL. Four studies reported planned radiographic measurements. The average number of levels fused in the included studies ranged from 6.4 to 14. The outcomes and follow-ups from each study are shown in Table 1.

**Table 1:** Summary of human studies using Medicrea patient-specific rods

Author, publication year	Country	Study design	Sample size	Pre-op measurements: Mean ± SD	Planned outcomes: Mean ± SD	Post-op outcomes: Mean ± SD	Follow-up, months post-op	Average levels fused ± SD
Barton et al., 2016	USA	Retrospective case series	18	SVA: 96.8 ± 56.8 mm PT: 32.0 ± 10.9° PI-LL: 29.2 ± 16.7°	SVA: 14.3 ± 22.4 mm PT: 20.5 ± 9.6° PI-LL: 0.9 ± 14.7°	SVA: 21.8 ± 37.8 mm PT: 17.7 ± 8.0° PI-LL: -4.1 ± 7.5°	1	11.4 ± 3
Solla et al., 2018	France	Prospective case series	60	No information provided	SVA: Preop to Planned *R*^2^ = .1(*p* = .317) PT: Pre-op to planned *R*^2^ = .2(*p* = .175) PI-LL: Pre-op to planned *R*^2^ = .1 (*p* = .505)	SVA: Pre-op to post-op *R*^2^ = .5 (*p* < .0001) PT: Pre-op to post-op *R*^2^ = .6(*p* < .001) PI-LL: Pre-op to post-op *R*^2^ = .5([< .0001)	12	6.4 (not provided)
Kleck et al., 2019	USA	Retrospective case series	34	SVA: 66.8 ± 48.2 mm PT:25.1 ± 9.7° PI-LL: 15.8 ± 17.4°	SVA: 9.9 ± 39.5 mm PT:15.2 ± 7.4° PI-LL:-2.6 ± 11.9°	SVA: 9.8 ± 33.9 mm PT: 23.2 ± 9.7° PI-LL: 1.6 ± 12.0°	34 patients 11–13 14 patients 23–25	10 ± 3.4
Prost and Farah et al., 2020 (A)	France	Prospective case series	86	Aligned SVA: 30.8 mm Non-Aligned SVA: 59.0 mm Aligned PT: 19.9° Nonaligned PT: 26.6° Aligned PI-LL: 4.1° Nonaligned PI-LL: 19.2°	No information provided	Aligned SVA: 0.7 mm Non-Aligned SVA: 41.2 mm Aligned PT: 18.3° Nonaligned PT: 27.0° Aligned PI-LL: -3.8° Nonaligned PI-LL: 11.6°	12	12.6 ± 3.6
Prost and Pesenti et al. 2020 (B)	France	Retrospective case series	77	SVA: 65.6 ± 65.7 mm PT: 26.3 ± 11.3° PI-LL: 18.1 ± 20.3°	No information provided	SVA: 38.5 ± 36.6 mm PT: 24.6 ± 11.9° PI-LL: 6.9 ± 11.8°	3	14.5 ± 3
Sadrameli et al., 2020	USA	Retrospective chart review	17	SVA: 65.7 ± 72.9 mm PT: 24.8 ± 9.6°	SVA: 14.8 ± 17.2 mm PT: 16.9 ± 3.8°	SVA: 21.6 ± 44.5 mm PT: 18.0 ± 8.6°	24	7.6 ± 2.7
Farah et al., 2022	France	Retrospective chart review	12	SVA: 58.2 mm PT: 31.7°	No information provided	SVA: 62.3 mm PT: 27.8°	12–70; mean 40.8	14 (not provided)

*SVA* sagittal vertical axis, *PT* pelvic tilt, *PI-LL* pelvic incidence—lumbar lordosis

### Sagittal vertical axis

Sagittal vertical axis (SVA) appeared as an outcome in seven papers. Few papers compared PSR’s predictive versus actual SVA. Barton performed a retrospective case series of 18 patients reporting overall SVA improvement (96.8 ± 56.8 mm to 21.8 ± 37.1; MD = 82.5 ± 14.40 mm, *p* < 0.001) but a significant difference was found between the projected outcome and the postoperative reality (14.3 ± 22.4 vs 21.8 ± 37.1, MD = 7.5 ± 43.3 mm, *p* = 0.002) [7].

In Solla et al., all 60 patients had post-operative goals of < 50 mm SVA, but 19 PSR patients retained SVA > 50 mm following surgery with PSR (*p* = *0*.5). Seven patients had a decrease yet remained above 50 mm, 6 had no decrease (uncorrected SVA), and 6 patients with initial SVA < 50 mm deteriorated to final SVA > 50 mm [8]. On linear regression analysis, preoperative SVA was associated with final SVA [*R* [2] = 0.5 (*p* < *0*.0001)], but the projected plan was not associated with final SVA [*R*^2^ = 0.1 (*p* = *0*.317)]. Preoperative SVA, pedicle subtraction osteotomy, and age were all significantly correlated with final SVA (*p* < 0.0001, *p* < *0*.0001, and *p* = *0*.021, respectively).

Sadrameli et al. retrospectively reviewed pre-operative, predicted, and post-operative spinopelvic parameters for a mixed treatment cohort of 34 patients, 17 with in situ bent rods versus 17 with PSRs [9]. There was no statistically significant difference between mean planned SVA of 14.8 ± 17.2 mm and post-operative SVA mean of 21.6 ± 44.5 mm (MD 6.8, p = 0.0045.) Of note, the mean pre-operative SVA was only moderately elevated at 65.65 mm in the cohort.

Kleck et al. reported an average improvement in SVA for their 34 patients from a mean of 66.8 ± 48.2 pre-operatively to a 2-year post-operative mean of 9.8 ± 33.9 (MD 57 ± 45.08 mm, p value not provided) [10]. The planned average of 9.9 ± 39.5 mm was almost identical to the actual final value (MD 0.1 ± 34.2 mm, p value not provided). Despite this, they found the predictive value of the plan was limited as sagittal balance was corrected more frequently than expected (*R*^2^ between 0.05 and 0.36 across follow-ups, *p* = 0.2).

Several studies report SVA improvement, but not compared to a projected plan. Prost et al. reported 11 of 86 patients with progressing SVA gain at 1-year post-surgery, although the entire cohort maintained statistically significant improvement compared to pre-operative measurements (MD 23 ± 8.1 mm, *p* = 0.007) [11]. 8 of 11 cases were due to PJK; they note revision surgery was not performed for these patients.

Prost and Pesenti later evaluated 77 patients at 3 months following ASD correction surgery involving PSRs, finding a similar improvement in SVA (mean 27.1 ± 8.6 mm, *p* < 0.0001), interestingly greater in patients with Parkinson’s disease (MD 53 mm, *p* < 0.005) [12]. Farah et al. also presented data on 12 patients with Parkinson’s disease, reporting improvement at 1 year follow-up (MD 76.1 mm, *P* = 0.013) [13].

### Pelvic incidence and lumbar lordosis

Barton in 2016 found for their 18 patients a significant difference in preoperative and postoperative PI-LL (29.2 ± 16.7° to -4.1 ± 7.5°; MD 33.3 ± 14.7°, *p* < 0.001). There was no significant MD between planned and postoperative PI-LL (0.9 ± 14.7° vs − 4.1 ± 7.5°; MD = 5.0 ± 13.8°, *p* = 0.147). There was a statistically significant correlation between the plan and postoperative PI-LL outcome (*R*^2^ = 0.4, *p* < 0.011).

Solla et al. found no significant correlations between planned versus actual PI-LL (*R*^2^ = 0.1, *p* = 0.5), although pre-operative PI-LL versus actual was significantly correlated (*R*^2^ = 0.5, *p* < *0*.0001). The influent factors on final PI-LL were found to be preoperative PI-LL and PSO (*p* < *0*.001). Age, sex, interbody fusion, number of levels, cage insertion, and SPO were not associated with final PI-LL (*p* > 0.1).

Kleck et al. captured weak to moderate predictive correlations between mean planned PI-LL of -2.6 ± 11.9° and the actual mean PI-LL of 1.6 ± 12.0° with weaker correlation at greater than 1-year follow-up (MD 4.2 ± 11.3°, *R*^2^ range 0.2 ≥ *R*^2^ ≤ 0.5, *p* ≤ 0.01). Overall improvement for PI-LL mismatch patients pre-operatively was noted, with 23 out of 34 patients (~ 67%) achieving less than 10° of mismatch one year (MD 14.2 ± 17.07°, *p* < 0.001).

Both studies published by Prost reported significant improvements in PI-LL but did not compare outcomes to a predicted plan. In Prost and Farah, 66 of the total 86 ASD patients had a preoperative PI-LL mismatch (15 ± 20°) and significant correction was achieved on average (8 ± 14°; MD 7°, *p* = 0.006). In Prost and Pesenti, they found that PI-LL decreased from 20.8° ± 17.8° to 8.3 ± 12.8° in the 43 ASD patients (MD 12.5°, *p* < 0.001).

Sadrameli and Farah included analysis only for LL and not PI-LL.

### Pelvic tilt

Barton et al. reported no significant difference between the planned mean pelvic tilt of 20.5 ± 9.6° and the post-operative pelvic tilt of 17.7 ± 8.0° (MD = 2.8 ± 7.7°, *p* = 0.144). However, linear regression analysis revealed no significant association between the plan and post-operative outcome (*R*^2^ 0.1, *p* = 0.174). PT did improve overall from a mean of 32 ± 10.9° to 17.7 ± 8.0° (MD = 14.3 ± 7.8°, *p* < 0.001).

Solla et al. similarly reported no significant correlation between the planned and post-operative PT [*R*^2^ = 0.2, (*p* = 0.175)]. Pre-operative PT instead better correlated with post-operative PT [*R*^2^ = 0.6, (*p* < 0.0001)]. Overall, four patients with PT > 20° decreased to < 20° and six were newly measured at > 20° post-operatively.

Kleck et al. conversely reports a moderate correlation between the plan and post-operative PT outcome [*R*^2^ = 0.4, (*P* < 0.02)]. The planned mean PT of 15.2 ± 7.4° was significantly different from the real mean PT of 23.2 ± 9.6° (MD 8° ± 10.5°, *p* < *0*.05). Long-term outcomes are unclear, however, due to 20 of the 34 original patients being lost to follow-up between the 1-year to 2-year interval.

Sadrameli noted that PT changes were not significantly different between surgery performed with PSRs versus operations completed using in situ rod bending (MD = 1.5°, *p* value not provided). PT did overall improve in the PSR group from 24.82 ± 9.6° to 18.00 ± 8.6° post-operatively (MD = 6.8, *p* < 0.01). The predicted PT of 16.9 ± 3.8° was similar to the actual post-operative alignment of 18.00 ± 8.6° (MD = 1.1°, *P* = 0.51).

PT was the least corrected of all spinopelvic parameters in Prost and Farah et al. There was no difference between the pre-operative mean and post-operative mean (*p* < 0.05), although nine cases newly fell outside normal range at the 1-year follow-up. The later Prost and Pesenti paper found no significant decrease in PT post-operatively overall for the entire cohort (MD = 1.7°, *p* = 0.154), or within the ASD-only group (MD = 1.5°, *p* = 0.437).

Farah observed a non-significant decrease in mean PT from 31.6° to 27.8° (MD = 3.8° *P* = 0.19) with an insignificant decrease to 28.9° at last follow-up (MD = 2.7°, *P* = 0.38).

### Other radiographic measures

Several additional measurements appeared in the literature. Barton et al. found sacral slope was similar between the planned and actual post-operative measurements (MD of 1.9 ± 8.4°, *p* = 0.376). The sacral slope post-operative–preoperative MD was 14.3 ± 8° (*p* < 0.001). Actual postoperative thoracic kyphosis was significantly different from the plan (MD = 8.1 ± 8.4°, *p* < 0.001). Barton also found a significant difference between planned mean thoracic kyphosis and actual mean post-operative thoracic kyphosis (MD of 8.1 ± 8.4°, *p* = 0.003).

Kleck measured mean post-operative sacral slope at 37.3 ± 12.9°, which was not statistically different from the planned mean of 42.5 ± 9.6° at the 2-year follow-up (MD = 5.2 ± 13.2°, *p* > 0.05). Pre-operative to post-operative sacral slope was not significantly different (MD 4.2 ± 14.6°, *p* > 0.05).

Sadrameli found no difference between plan and outcome for sacral slope (MD = 0.8 ± 10.7°, *p* = 0.62). There was a significant change from pre-operative sacral slope to post-operative sacral slope (MD 7.9 ± 7.8°, *p* = 0.0006). Additionally, Sadrameli examined blood loss and operative time. They found no statistical difference in the blood loss between the PSR rod group (861 ± 354 cc) and traditional rod group (913 ± 111 cc, MD 52 ± 141.3 cc, *p* = 0.35). There was also no significant difference in the mean PSR operative time of 411 ± 93 min versus the traditional rod operative time of 421 ± 111 min (MD 10 ± 64, min *p* = 0.76).

### Complications and reoperations

Barton and Sandrameli reported neither complications nor reoperations.

Kleck and colleagues reported a 58.8% one-time complication rate and 38.2% two-plus rate. Originally, the cohort included 43 patients, but when 9 patients required revision surgery during the second post-operative year they were excluded from the analysis. Proximal junctional failure, which occurred in four patients, was the most common reason for revision. Two vertebral fractures and two instances of screw loosening required revision. One patient underwent revision due to development of postoperative sagittal imbalance.

Prost and Farah reported a major complication rate of 30.2% of 86 patients at 1-year follow-up. A total of 11 patients required re-operation, 8 due to rod breakage and 3 for proximal junctional kyphosis requiring an extension of the fusion.

Prost and Pesenti note that 4% (*n* = 3) of their patients had mechanical complications (proximal junctional kyphosis) at 3-month post-operative. In Farah et al., revision surgery was required for 8 of the 12 Parkinson’s disease patients with 6 due to rod breakage and 2 due to surgical site infections. Solla noted five patients underwent revision for persistent imbalance but that there were no rod fractures.

### Quality assessment

Overall, a moderate risk of bias was found among the studies included in this review. Three studies, Barton et al., Prost and Pesenti, and Solla were identified as possessing moderate post-interventional risk for bias due follow-up less than 12 months. In general, 12 months is considered the threshold at which reliable deformity correction data may be reported, and the optimal minimum for obtaining reliable follow-up data is 2 years [14] It is possible that short-term follow-up overestimates the efficacy of PSRs.

A moderate risk of pre-interventional bias was identified in two studies—Prost and Pesenti and Farah—as they included patients with Parkinson’s Disease in their studies. These patients exhibit a predilection for less favorable deformity correction results as Parkinson’s disease is associated with significantly higher complications and reoperation rates. Additionally, the results of these studies are questionable due to high complication rates and the loss of numerous patients to follow-up. In Prost and Farah’s study, for example, nine patients were excluded from the 2-year follow-up due to their undergoing revision surgery. The remainder of the studies included in the present review were determined to be of low pre-interventional, interventional, and post-interventional risk of bias.

## Discussion

SVA, PI-LL, and PT are critical spino-pelvic parameters used to assess the severity of ASD [15, 16]. Failure to reach values of SVA < 40–50 mm, PT < 20°, and PI-LL < 10° is correlated with significant mechanical complications such as PJK or pseudarthrosis while obtaining appropriate alignment is correlated with improvement of patient outcome [17–20]. Though useful, there is significant room for improvement in using these parameters as their correlation with patient-reported outcomes can be relatively weak [21]. In an effort to address this, surgeons have begun to create patient-specific goals that take into account other factors including the age of the patient, the Roussouly four-type sagittal shape, and GAP score [1, 22–26]. However, the papers included in this review primarily report SVA, PI-LL, and PT goals in the classic fashion with static goals for all patients (SVA < 40° or < 50°, PI-LL < 10°, and PT < 20°). Studies on PSRs in the future should incorporate more modern techniques for planning and individualized patient radiographic goals into their study design.

In this review, all studies except Solla 2018 reported significant improvement in SVA postoperatively. Rates of SVA correction with traditional operative correction techniques have variable success rates and thresholds for what is considered correction vary by study. A multicenter prospective study found that ~ 50% of patients at 1-year post-operative follow-up exhibited SVAs within acceptable ranges (SVA < 40 mm) [27]. Kleck found that using PSRs 72% of patients reached that goal of SVA < 40 mm. The five studies reporting PI-LL and the two reporting LL demonstrated significant postoperative improvement. The study by Barton et al. showed that all patients achieved an optimal PI-LL range of less than 10° at their last follow-up and Solla found that 66% of patients who initially had a PI-LL above 10° ultimately reached this optimal range. For comparison, a previous retrospective cohort study of 164 patients using traditional rods for ASD reported only 51% of patients had a PI-LL < 10 post-operatively [28]. Three of seven studies found a significant improvement in average postoperative PT including Kleck, in which the improvement was present in approximately 25% of patients. Solla, Sadrameli, and Farah found a non-significant improvement while Prost and Farah found no improvement at all.

Studies have shown that correcting the pelvic tilt is more difficult [29, 30] Despite this, the Parkinson’s patients in Prost and Pesenti did have a significant improvement in pelvic tilt. These results in conjunction with the side-to-side comparison to traditional rods in Sadrameli et al., indicate that PSRs are effective in correcting alignment with potential to be superior, but it is far from conclusive evidence for either non-inferiority or superiority. There have been no randomized controlled trials or matched cohort head-to-head comparisons to date, although a double-blind randomized trial is in progress [31].

Whether using traditional or patient-specific rods, pre-operative goals for alignment are necessary to define degree of correction desired intraoperatively [32, 33]. Only four studies included an analysis comparing postoperative and planned outcomes. Barton and Sadrameli found a significant association between preoperative plan and postoperative outcomes in all three measures. Kleck found a significant association between prediction and outcome for PI-LL alone. Solla found no significant correlation between their plans and post-operative outcomes. From these studies, the effect of PSRs on alignment achieved remains unclear and it is vital for future studies on PSRs to include planned outcomes for each patient so a true evaluation of their potential benefit may be conducted.

Proponents of the patient-specific rods postulate that removing the perioperative bending step will reduce operation time and consequentially blood loss [5]. Sadramelli et al., is the only paper that examined these factors, finding a small 10-min reduction in operative time and 50 mL less blood loss for the PSR group. The differences were not statistically significant nor likely clinically significant. With only one study examining these factors, the results are far from conclusive.

Revision rates are documented anywhere from 9 to 45% for ASD operations with a national analysis between 2005 and 2011 estimated an 18% incidence at 4 years post-op [19, 28–31]. Kleck excluded 20% (9 of 43) of the original patients in their study as they required revision before the second year (4 for PJK). The study did not report the non-revised incidence of PJK [37]. Additionally, 20 more patients were lost to follow-up prior to the second year, which may further confound re-operation rate. Prost and Farah reported a lower ~ 13% (11 of 86) reoperation rate, while Prost and Pesenti reported no reoperations but a 4% (3 of 77) rate of PJK. These studies had short follow-up times of 1 year and 3 months, respectively. Follow-up was in general limited in our review. Three studies included patients with follow-up times ≤ 24 months with very few patients reaching 24 months. Farah et al. had the longest follow-up with an average of 40 months and the highest re-operation rate of ~ 66% (8 of 12). The study included Parkinsonian patients, which is likely associated with this incidence [29, 38, 39].In addition to the profound effects of revisions on patient-wellbeing, revision surgery represents a major portion of the cost-burden in treating ASD patients and increases the average treatment cost by up to 70% [40, 41]. Further study of the PSR revision rates is a pre-requisite for an accurate economic comparison to traditional methods of ASD treatment.

Prevalence of revision surgery was reported in our review and the most common reason for revision is rod fracture/instrument failure [19, 42, 43]. PSRs have been advertised to have a fracture rate as low as 2.2% compared to the traditional rate of 15% as found in their white paper [44]. Included studies found a wide range of rod fracture rates with Solla reporting 0% (0 of 60), Prost and Farah 9% (8 of 86) and Farah 50% (6 of 12) indicating that there may be significant confounding factors, including the ASD etiology. The lack of reporting on complications in two studies, the limited follow-up, and the small sample sizes prevent adequate comparisons between values in the literature and the results of included studies. An accurate rod fracture rate comparison is crucial for determining non-superiority of patient-specific rods and performing a cost–benefit analysis between the two treatments.

Overall, the analysis of current literature on ASD surgery with patient-specific rods is hindered by confounding variables affecting grouped patient data. The heterogeneity of surgical methods, surgical tools, length of fusion, and pre-operative diagnoses used for ASD correction may serve as significant confounding variables for both intra- and inter-study comparisons. Patients in these studies underwent operations including, but not limited to, Smith-Peterson osteotomy (SPO), pedicle subtraction osteotomy (PSO), vertical column resection (VCR), anterior lumbar interbody fusion (ALIF), and posterior lumbar interbody fusion (PLIF). The significance of this variation can be observed in the study performed by Solla and colleagues, who found that patients undergoing PSO were 3.8 times more likely to show improvement in all three alignment parameters at 1 year follow-up (*p* = 0.03). Further, all rods were from the same manufacturer, but not all the rods were the same material. Barton, Solla, and Farah used titanium rods, Kleck and Prost and Pesenti did not specify, Prost and Farah used titanium rods for 47 patients with cobalt chromium rods for the other 39 and Sadrameli used all cobalt chromium rods. The cobalt chromium rods are stiffer and believed to obtain higher rates of deformity correction but associated with an increased risk of proximal junctional kyphosis [45]. Construct properties such as screw type/number/placement, hooks, and cross connectors were only reported in one included manuscript despite being known to affect complication rates [46]. Prost et al., reported using poly axial pedicle screws in the thoracolumbar area, with an unspecified number of cases having sub-laminar hooks placed at the upper instrumented level when pedicle screw insertion was impossible. The number of levels fused also constitutes a significant difference in surgical techniques between studies. As an example, Solla et al. reported only half as many levels fused on average when compared to Farah et al.

In addition to surgical technique employed, there was significant heterogeneity in the wide range of etiologies of ASD included in the present study. These included but were not limited to Parkinson’s disease, sagittal imbalance resulting from degenerative change, post-traumatic kyphosis, idiopathic flat back syndrome, and ankylosing spondylitis. Notably, Farah included only patients with both ASD and Parkinson’s disease while the Prost and Farah paper included a subset of 10 patients with both ASD and Parkinson’s disease as well as two other subgroups. As previously stated, Parkinson’s patients are more difficult to treat and have higher complication rates resulting from their specific ASD etiology. Future studies isolating subsets of patients with specific etiologies, operations, and construct types, or individualized patient data for meta-analysis are crucial to increase the measurable predictive value of the preoperative plans and allow for stronger comparisons between studies.

## Conclusions

The current literature provides some evidence for the efficacy of PSRs in ASD surgery, particularly in achieving optimal spino-pelvic parameters including SVA, PI-LL, and PT. However, these findings must be interpreted with caution due to the heterogeneity of enrolled patients, lack of randomized controlled trials, and the considerable interplay of confounding variables such as surgical techniques, ASD etiologies, construct properties and rod materials. While PSRs hold potential for improved pre-operative planning and reduced operation time and blood loss, the evidence is not conclusive. Furthermore, the impact of PSRs on revision rates and rod fractures remains unclear due to the presence of confounders such as differing follow-up time and patient populations between studies. While PSRs may offer potential benefits, a lack of robust, high-quality evidence, combined with the considerable variation in study parameters, precludes definitive conclusions about their superiority over traditional rods. Future research should aim to improve pre-operative planning, isolate specific patient subgroups, employ standardized methodologies, and establish head-to-head comparisons with traditional rods to better understand the true impact of PSRs in ASD surgery.

## Data Availability

Data available upon request.
